# Extracorporeal Membrane Oxygenation Candidacy in Pediatric Patients Treated With Hematopoietic Stem Cell Transplant and Chimeric Antigen Receptor T-Cell Therapy: An International Survey

**DOI:** 10.3389/fonc.2021.798236

**Published:** 2021-12-22

**Authors:** Saad Ghafoor, Kimberly Fan, Matteo Di Nardo, Aimee C. Talleur, Arun Saini, Renee M. Potera, Leslie Lehmann, Gail Annich, Fang Wang, Jennifer McArthur, Hitesh Sandhu

**Affiliations:** ^1^ Department of Pediatric Medicine, Division of Critical Care, St. Jude Children’s Research Hospital, Memphis, TN, United States; ^2^ Division of Pediatric Critical Care, University of Tennessee (IT) Health Science Center, Memphis, TN, United States; ^3^ Pediatric Intensive Care Unit, Bambino Gesù Children’s Hospital, Istituto di Ricovero e Cura a Carattere Scientifico (IRCCS), Rome, Italy; ^4^ Department of Bone Marrow Transplant and Cellular Therapy, St. Jude Children’s Research Hospital, Memphis, TN, United States; ^5^ Division of Pediatric Critical Care, Texas Children’s Hospital and Baylor College of Medicine, Houston, TX, United States; ^6^ Division of Pediatric Critical Care, University of Tennessee (UT) Southwestern Medical Center, Dallas, TX, United States; ^7^ Pediatric Hematology-Oncology, Dana Farber Boston Children’s Cancer and Blood Disorder Center, Boston, MA, United States; ^8^ Department of Critical Care Medicine, University of Toronto/The Hospital for Sick Children, Toronto, ON, Canada; ^9^ Department of Biostatistics, St. Jude Children’s Research Hospital, Memphis, TN, United States; ^10^ Division of Pediatric Critical Care, Medical College of Wisconsin, Milwaukee, WI, United States

**Keywords:** extracorporeal membrane oxyenation, extracorporeal life support (ECLS), hematopoeietic cell transplant, chimeric antigen receptor T-cell (CAR-T) therapy, pediatric oncology, pediatric critical care, onco-critical care

## Abstract

**Introduction:**

Pediatric patients who undergo hematopoietic cell transplant (HCT) or chimeric antigen receptor T-cell (CAR-T) therapy are at high risk for complications leading to organ failure and the need for critical care resources. Extracorporeal membrane oxygenation (ECMO) is a supportive modality that is used for cardiac and respiratory failure refractory to conventional therapies. While the use of ECMO is increasing for patients who receive HCT, candidacy for these patients remains controversial. We therefore surveyed pediatric critical care and HCT providers across North America and Europe to evaluate current provider opinions and decision-making and institutional practices regarding ECMO use for patients treated with HCT or CAR-T.

**Methods:**

An electronic twenty-eight question survey was distributed to pediatric critical care and HCT providers practicing in North America (United States and Canada) and Europe through the Pediatric Acute Lung Injury and Sepsis Investigators (PALISI) Network and individual emails. Responses to the survey were recorded in a REDCap^®^ database.

**Results:**

Two-hundred and ten participants completed the survey. Of these, 159 (76%) identified themselves as pediatric critical care physicians and 47 (22%) as pediatric HCT physicians or oncologists. The majority (99.5%) of survey respondents stated that they would consider patients treated with HCT or CAR-T therapy as candidates for ECMO support. However, pediatric critical care physicians identified more absolute and relative contraindications for ECMO than non-pediatric critical care physicians. While only 0.5% of respondents reported that they consider HCT as an absolute contraindication for ECMO, 6% of respondents stated that ECMO is contraindicated in HCT patients within their institution and only 23% have an institutional protocol or policy to guide the evaluation for ECMO candidacy of these patients. Almost half (49.1%) of respondents would accept a survival to hospital discharge of 20-30% for pediatric HCT patients requiring ECMO as adequate.

**Conclusions:**

ECMO use for pediatric patients treated with HCT and CAR-T therapy is generally acceptable amongst physicians. However, there are differences in the evaluation and decision-making regarding ECMO candidacy amongst providers across medical specialties and institutions. Therefore, multidisciplinary collaboration is an essential component in establishing practice guidelines and advancing ECMO outcomes for these patients.

## Introduction

Pediatric and young adult patients who receive hematopoietic cell transplant (HCT) are at high risk for multi-organ dysfunction and need for critical care support. Analyses of the Extracorporeal Life Support Organization (ELSO) database from 1991-2012 show dismal outcomes for pediatric HCT patients who require extracorporeal membrane oxygenation (ECMO) support, with only a 5-10% survival to hospital discharge (1–3). Given the poor outcomes, ECMO use for pediatric HCT patients is controversial and many consider HCT as a contraindication for ECMO (4–9). However, the indications for ECMO have evolved since its introduction in the 1980s with increasing use in septic shock, trauma, and pulmonary hemorrhage, conditions which were once considered contraindications. Additionally, significant advancements in HCT conditioning regimens, infection control, supportive care, mechanical ventilation, and ECMO technology have led to improved outcomes for both HCT and ECMO patients (10–14). Over the last decade, recognizing these advances and the knowledge gained from the care of critically ill pediatric HCT patients, some centers have reported successful ECMO use in a certain HCT patients (15–19). These reports have led to a renewal of interest and suggest improving outcomes for pediatric HCT patients supported with ECMO. This is also supported by recent reports that demonstrate improved survival of 26-50% for pediatric HCT patients supported with ECMO since 2010 (20, 21).

Chimeric antigen receptor T-cell (CAR-T) therapy is a novel treatment option, offering high chance of remission for a historically incurable patient population, particularly those with relapsed or refractory leukemia (22–25). However, CAR-T therapy comes with the risk of severe acute complications due to systemic inflammatory responses (including cytokine release syndrome [CRS], immune effector cell-associated neurotoxicity [ICANS], and CAR-T associated hemophagocytic lymphohistiocytosis [carHLH]), rapid tumor cell death, and infection, which can lead to cardiac and/or respiratory failure (22, 26–29). While to our knowledge, there has been no reports of ECMO for post CAR-T therapy complications in pediatrics, Stoner, et al, reported a case of successful veno-arterial ECMO use in a pediatric patient with septic shock as a bridge to CAR-T (30).

Given the global improvements in the care of pediatric HCT patients and increasing use of CAR-T therapy, we surveyed providers across North America and Europe to evaluate the current opinions and practice regarding ECMO use for pediatric patients treated with HCT and CAR-T therapy. We also sought to assess the contribution of various patient- and disease-related factors to ECMO candidacy. Further, as providing care for critically ill patients treated with HCT and CAR-T therapy requires provider input across multiple disciplines, we aimed to evaluate differences in decision-making between providers across specialties in order to identify ways to improve ECMO access for this vulnerable patient population.

## Materials and Methods

### Survey Development

We formed a multidisciplinary committee consisting of seven pediatric critical care physicians, including one extracorporeal life support program director, and two bone marrow transplant/cellular therapy physicians to develop a 28-question survey to evaluate three major content areas: (1) physician and institutional experience in caring for pediatric HCT and ECMO patients; (2) provider opinions and institutional practices regarding the use of ECMO support for pediatric HCT patients; and (3) provider opinions and institutional practices regarding the use of ECMO support for pediatric CAR-T patients (**Supplemental Figure 1**). The survey was distributed to a pilot group of pediatric critical care and HCT providers for review and revised according to feedback prior to study initiation (31).

### Survey Distribution and Data Collection

The survey was distributed electronically to pediatric critical care, pediatric bone marrow transplant and cellular therapy, and pediatric oncology physicians at institutions across North America and Europe. Included institutions were identified by the authors as centers providing greater than 20 pediatric allogeneic HCT/year, using publicly available data. Email addresses for physicians were obtained through hospital or organizational websites. The survey was also approved and distributed to members of the Pediatric Acute Lung Injury and Sepsis Investigators (PALISI) Network. Study subjects were emailed an introduction to the study and link to the survey. The survey was administered, and data stored anonymously on a Red Cap database. All participants were given 56 days to complete the survey with reminders sent after 14, 28, and 41 days to individual participants and after 1 month to PALISI members. Consent to participate was implied with completion of the survey. The study was reviewed and approved by the St. Jude Children’s Research Hospital Institutional Review Board.

### Statistical Analysis

The Chi-square test was used to compare the differences in survey responses between groups: (1) pediatric critical care medicine (PCCM) and non-PCCM providers, (2) respondents in centers located in North America and Europe, (3) respondents in high-volume and low-volume ECMO centers, (4) respondents in high-volume and low-volume HCT centers, and (5) respondents in centers with and without a protocol for ECMO support in HCT patients. For items with less than 5 expected responses, the Fisher’s Exact test was used instead of the Chi-square test to ensure statistical validity. Normality was tested for the total number of answers selected using the Shapiro-Wilk test. For non-normally distributed data, the Mann Whitney U test was conducted to compare the group difference. All analyses were performed using SAS software, version 9.4, Copyright (c) 2016 by SAS Institute Inc., Cary, NC, USA.

## Results

### Survey Respondents

Our REDCap^®^ database recorded a total of 511 survey invites. Of these, 244 (47.7%) respondents started the survey and 210 (41.1%) completed the survey. Of the respondents who completed the survey, 159 (76%) identified themselves as a pediatric critical care physician and 39 (19%) reported that they are their institution’s ECMO director. Forty-seven (22%) respondents identified themselves as a pediatric HCT physician or pediatric oncologist providing CAR-T therapy. Regionally, 184 (88%) respondents practice in the United States, 7 (3%) in Canada, and 19 (9%) in Europe with the majority (95%) working in an academic medical center. One hundred and seventy-one (81%) respondents provided their current institution and 39 (19%) preferred not to answer. Of those who provided their current institution, there were 69 identified institutions. Within the United States, participants identified 12 institutions in the Northeast (24 respondents), 14 institutions in the Midwest (44 respondents), 7 institutions in the West (31 respondents), and 17 institutions in the South (50 respondents). There were 3 identified institutions in Canada (4 respondents) and 16 institutions identified in Europe (18 respondents). Most respondents, 201 (96%), reported that they provide medical care for pediatric HCT patients and 103 (49%) stated that their institution performs more than 30 HCTs per year. 186 (89%) of respondents reported that their institution provides care to patients who have received or are receiving CAR-T therapy (**Table 1**).

**Table 1:** Respondent characteristics.

Variable		N (%)
Total Respondents		210
Medical Specialty		
Pediatric critical care medicine		159 (75.7)
HCT/Oncology		47 (22.4)
Other		4 (1.9)
Years of Experience		
0-5		47 (22.4)
6-10		52 (24.7)
11-20		65 (31)
>20		46 (21.9)
ECMO Director		
Yes		39 (18.6)
No		171 (81.4)
Organization Membership		
PALISI		165
PALISI- HCT Subgroup		79
ELSO		140
PBMTC		65
None of the above		11
Institution Location		
United States		184 (87.6)
Canada		7 (3.3)
Europe		19 (9.1)
Institution Type		
Academic center		199 (94.8)
Private practice		0 (0)
Government Hospital		8 (3.8)
Other		3 (1.4)
Institutional ECMO Runs (per year)		
0-10		50 (23.8)
11-30		58 (27.6)
>30		66 (31.4)
Unsure		36 (17.2)
Institutional HCT Performed (per year)		
0-10		20 (9.5)
11-30		18 (8.6)
>30		103 (49)
Unsure		69 (32.9)
Institutional # of HCT ICU admissions (per year)		
0-20		73 (34.8)
21-50		42 (20)
>50		58 (27.6)
Unsure		37 (17.6)

HCT, hematopoietic cell transplant; ECMO, extracorporeal membrane oxygenation; PALISI, Pediatric Acute Lung Injury and Sepsis Investigators; ELSO, Extracorporeal Life Support Organization; PBMTC, Pediatric Bone and Marrow Transplant Consortium; ICU, intensive care unit.

### ECMO Use in Patients Undergoing HCT

The majority of respondents (99.5%) stated that they would consider pediatric HCT patients as candidates for ECMO. Specifically, 95.7% of respondents reported that they think every HCT patient is unique thus candidacy for ECMO should be considered on an individual basis. Only 3.8% responded that they think the criteria for ECMO is the same between HCT and other pediatric patients and 0.5% think of HCT as an absolute contraindication for ECMO. Respondents cited the following sources as contributing to their opinion regarding ECMO candidacy for HCT patients: historical outcomes data (24.8%), personal experience (23.8%), institutional experience or policy (27.2%), current standard of practice in their region/country (17.1%), or another source (7.1%). All respondents reported that they would find a survival to hospital discharge < 60% as acceptable for pediatric HCT patients supported with ECMO, with 49.1% accepting 20%-30% and 29.5% accepting 30%-40% survival to hospital discharge for this patient population. (**Table 2**).

**Table 2:** Provider opinion on extracorporeal membrane oxygenation candidacy for pediatric patients treated with hematopoietic cell transplant.

Question	Response	N (%)
Which statement best fits your opinion regarding the use of ECMO in pediatric HCT patients?	HCT is an absolute contraindication for ECMO.	1 (0.5)
	Every HCT patient is unique thus candidacy should be considered on an individual basis.	201 (95.7)
	ECMO criteria for HCT patients is the same as for any other patient.	8 (3.8)
What source contributes most to your opinion regarding ECMO candidacy for HCT patients?	Historical data on outcomes	52 (24.8)
	Past personal experience	50 (23.8)
	Institutional experience	47 (22.4)
	Institutional policy/protocol	10 (4.8)
	Current standard of practice in region/country	36 (17.1)
	Other	15 (7.1)
What is an acceptable rate of survival to hospital discharge for HCT patients requiring ECMO support?	20%-30%	103 (49.1)
	30%-40%	62 (29.5)
	40%-50%	36 (17.1)
	50%-60%	9 (4.3)
	>60%	0 (0)
What is the minimum acceptable level of evidence to establish ECMO candidacy for HCT patients?	Case report/series	39 (18.6)
	Registry reports	62 (29.5)
	Expert consensus statement	83 (39.5)
	Clinical trial	15 (7.2)
	No further studies needed	11 (5.2)

ECMO, extracorporeal membrane oxygenation; HCT, hematopoietic cell transplant.

On an institution basis, 80% of respondents reported that their institution considers ECMO candidacy for HCT patients individually while 6% of respondents reported that their institution considers HCT as an absolute contraindication for ECMO. Only 48 (23%) respondents reported that their institution has a protocol or policy to address ECMO candidacy for these patients, while 162 (77%) respondents stated that their institution either does not have or were unsure if they had such a protocol/policy. Discussion regarding ECMO candidacy involves input from multiple disciplines including the on-call intensivist (96.2%), on-call HCT physician (79%), patients primary HCT physician (58.6%), ECMO director/consult team (82.4%), and cannulating surgeon (61.4%). However, consensus regarding ECMO candidacy amongst teams was often variable. Between the critical care and HCT teams, 18.1% of respondents reported that there is ‘always’ consensus and 64.3% reported that there is ‘sometimes’ consensus. Between the critical care and ECMO consult team, 38.1% of respondents reported that there is ‘always’ consensus and 46.6% reported that there is ‘sometimes’ consensus regarding candidacy for ECMO support. (**Table 3**). To influence provider opinion towards ECMO candidacy in these patients, 39.5% of respondents would look towards expert/committee consensus statements, 29.5% further registry reports, 18.6% case reports/series, 7.2% clinical trials, and 5.2% feel that no further studies are needed on this topic (**Table 2**).

**Table 3:** Institutional practice for extracorporeal membrane oxygenation use for patients treated with hematopoietic cell transplant.

Question	Response	N (%)
Which statement best fits your institution’s practice regarding ECMO use in HCT patients?	HCT is an absolute contraindication for ECMO.	12 (5.7)
	Every HCT patient is unique thus candidacy for ECMO should be considered on an individual basis.	167 (79.5)
	ECMO criteria for HCT patients is the same as for any other patient.	4 (1.9)
	No consensus.	27 (12.9)
Have any post-HCT patients received ECMO support at your institution? If yes, how many (last 5 years)?	No	53 (25.3)
	Yes, 1 to 5	104 (49.5)
	Yes, >5	16 (7.6)
	Unsure	37 (17.6)
Does your institution have a protocol/policy to determine HCT ECMO candidacy?	Yes	48 (22.9)
	No	142 (67.6)
	Unsure	20 (9.5)
Physicians/teams participating in ECMO candidacy decision-making for HCT patients:	Intensivist on-call/service	202 (96.2)
	BMT physician on-call/service	166 (79)
	Primary BMT physician	123 (58.6)
	ECMO director/consult team	173 (82.4)
	Cannulating surgeon	129 (61.4)
Institutionally, how often is there consensus between the following medical teams regarding ECMO candidacy for an HCT patient?	Individual intensivist	
	Always	59 (28.1)
	Sometimes	125 (59.5)
	Never	2 (1)
	Unsure	24 (11.4)
	Critical care and HCT	
	Always	38 (18.1)
	Sometimes	135 (64.3)
	Never	16 (7.6)
	Unsure	21 (10)
	Critical care and surgery	
	Always	56 (26.7)
	Sometimes	113 (53.8)
	Never	5 (2.4)
	Unsure	36 (17.1)
	Critical care and ECMO consult team	
	Always	80 (38.1)
	Sometimes	98 (46.6)
	Never	2 (1)
	Unsure	30 (14.3)

ECMO, extracorporeal membrane oxygenation; HCT, hematopoietic cell transplant; ICU, intensive care unit.

We evaluated 15 patient and treatment related factors that may influence ECMO candidacy and asked participants to identify factors that they would consider as absolute or relative contraindications for ECMO in the HCT patient (**Table 4**). Of these, the most selected absolute contraindication was the presence of multiple organ failure (54%), followed by an expected 1-year survival <50% from the underlying disease (38%) and active pulmonary hemorrhage (37%). The most selected relative contraindication was a length of mechanical ventilation > 14 days (34%), followed by refractory thrombocytopenia (33%) and active pulmonary hemorrhage (33%).

**Table 4:** Respondent selections of absolute and relative contraindications for extracorporeal membrane oxygenation in pediatric patients treated with hematopoietic cell transplant.

Factor	Absolute Contraindication		Relative Contraindication	
	N (%)	p-value^#^	N (%)	p-value^#^
Allogeneic HCT				
Overall (N=210)	1 (0.5)		13 (6.2)	
PCCM (N=159)	1 (0.6)	1	13 (8.2)	0.035
Non-PCCM (N=51)	0 (0)		0 (0)	
Autologous HCT				
Overall	0 (0)		4 (1.9)	
PCCM	0 (0)	—	4 (2.5)	0.574
Non-PCCM	0 (0)		0 (0)	
Multiple organ failure				
Overall	114 (54.3)		61 (29)	
PCCM	87 (54.7)	0.825	43 (27)	0.259
Non-PCCM	27 (52.9)		18 (35.3)	
Expected 1-year survival < 50% from underlying disease				
Overall	79 (37.6)		66 (31.4)	
PCCM	74 (46.5)	<0.001	54 (34)	0.163
Non-PCCM	5 (9.8)		12 (23.5)	
Active pulmonary hemorrhage				
Overall	77 (36.7)		69 (32.9)	
PCCM	61 (38.4)	0.367	59 (37.1)	0.021
Non-PCCM	16 (31.4)		10 (19.6)	
Secondary graft failure				
Overall	73 (34.8)		50 (23.8)	
PCCM	58 (36.5)	0.357	34 (21.4)	0.145
Non-PCCM	15 (29.4)		16 (31.4)	
Refractory thrombocytopenia				
Overall	70 (33.3)		70 (33.3)	
PCCM	58 (36.5)	0.088	56 (35.2)	0.306
Non-PCCM	12 (23.5)		14 (27.5)	
Mechanical ventilation > 14 days				
Overall	53 (25.2)		72 (34.3)	
PCCM	45 (28.3)	0.071	62 (39)	0.011
Non-PCCM	8 (15.7)		10 (19.6)	
GVHD, grade III or higher				
Overall	43 (20.5)		59 (28.1)	
PCCM	37 (23.3)	0.076	48 (30.2)	0.233
Non-PCCM	6 (11.8)		11 (21.6)	
Pre-engraftment				
Overall	39 (18.6)		38 (18.1)	
PCCM	35 (22)	0.024	31 (19.5)	0.352
Non-PCCM	4 (7.8)		7 (13.7)	
VOD/SOS				
Overall	38 (18.1)		54 (25.7)	
PCCM	33 (20.8)	0.077	45 (28.3)	0.130
Non-PCCM	5 (9.8)		9 (17.7)	
≥ 2 HCT				
Overall	37 (17.6)		55 (26.2)	
PCCM	33 (20.8)	0.035	42 (26.4)	0.896
Non-PCCM	4 (7.8)		13 (25.5)	
Unknown etiology of decompensation				
Overall	32 (15.2)		60 (28.6)	
PCCM	25 (15.7)	0.730	51 (32.1)	0.047
Non-PCCM	7 (13.7)		9 (17.7)	
HCT < +100 days				
Overall	8 (3.8)		34 (16.2)	
PCCM	8 (5)	0.102	32 (20.1)	0.006
Non-PCCM	0 (0)		2 (3.9)	
Non-oncologic disease as reason for transplant				
Overall	0 (0)		7 (3.3)	
PCCM	0 (0)	—	6 (3.8)	0.530
Non-PCCM	0 (0)		1 (2)	

HCT, hematopoietic cell transplant; PCCM, pediatric critical care medicine; GVHD, graft versus host disease; VOD, veno-occlusive disease; SOS, sinusoidal obstruction syndrome. ^#^p-value reflects comparison between PCCM and non-PCCM responses.

Pediatric critical care medicine (PCCM) providers selected significantly more factors as absolute contraindications (3 [IQR 3] *vs* 2 [IQR 2], p<0.001) and relative contraindications (3 [IQR 2] *vs* 2 [IQR 3], p=0.002) compared to non-PCCM providers (**Supplemental Table 1**). There was no significant difference in the number of factors selected as absolute contraindications between respondents from North America (United States and Canada) versus Europe, high volume (≥30/year) versus low volume (<30/year) ECMO centers, high volume (≥30/year) versus low volume (<30/year) HCT centers, or centers with an HCT ECMO protocol versus those without a HCT ECMO protocol. Similarly, there was no significant difference in the number of relative contraindications selected between respondents from North American versus Europe, high volume versus low volume ECMO centers, high volume versus low volume HCT centers, or centers with an HCT ECMO protocol versus those without a HCT ECMO protocol.

Compared to non-PCCM providers, significantly more PCCM providers selected the need for two or greater HCTs (20.8% vs 7.8%, p=0.035), pre-engraftment (22% *vs* 7.8%, p=0.024), and an expected 1-year survival < 50% due to underlying disease (46.6% vs 9.8%, p<0.001) as absolute contraindications for ECMO. PCCM providers were also significantly more likely to select allogeneic transplant (8.2% *vs* 0%, p=0.035), less than 100 days since HCT (20.1% *vs* 3.9%, p=0.006), active pulmonary hemorrhage (37.1% *vs* 19.6%, p=0.021), and an unknown etiology for decompensation (32.1% vs 17.7%, p=0.047) as relative contraindications for ECMO (**Table 4**).

### ECMO Use in Patients Undergoing CAR-T Therapy

Overall, 132 (62.8%) respondents think ECMO candidacy for patients undergoing CAR-T therapy and HCT are very different thus candidacy should be evaluated on an individual basis, 77 (36.7%) respondents think ECMO candidacy should be evaluated similarly, and 1 (0.5%) respondent considered CAR-T therapy as an absolute contraindication for ECMO (**Table 5**). Respondents cited the following sources as contributing most to their opinion regarding ECMO candidacy for pediatric patients treated with CAR-T therapy: personal experience (25.2%), institutional experience or policy (28.1%), current standard of practice in their region/country (35.7%), or another source (11%).

**Table 5:** Provider opinion regarding use of extracorporeal membrane oxygenation for patients treated with chimeric antigen receptor t-cell therapy.

Question	Response	N (%)
Does your center care for patients who have received CAR-T therapy?	Yes	186 (88.6)
	No	16 (7.6)
	Unsure	8 (3.8)
Which statement best fits your opinion regarding use of ECMO in pediatric CAR-T patients?	ECMO for CAR-T and HCT patients should be evaluated similarly.	77 (36.7)
	ECMO for CAR-T and HCT are very different and should be evaluated individually.	132 (62.9)
	Undergoing treatment with CAR-T is an absolute contraindication for ECMO.	1 (0.4)
Which statement best fits your institution’s practice regarding use of ECMO in pediatric CAR-T patients?	ECMO for CAR-T and HCT patients should be evaluated similarly.	50 (23.8)
	ECMO for CAR-T and HCT are very different and should be evaluated individually.	72 (34.3)
	Undergoing treatment with CAR-T is an absolute contraindication for ECMO.	0 (0)
	No consensus.	62 (29.5)
	Unsure	26 (12.4)
Which source contributes the most to your opinion on ECMO candidacy in CAR-T?	Personal opinion based on past experience	53 (25.2)
	Institutional experience	51 (24.3)
	Institutional policy/practice	8 (3.8)
	Current standard practice in region/country	75 (35.7)
	Other	23 (11)
What is your opinion regarding ECMO candidacy for CAR-T patients in each of the following circumstances:		
1st CAR-T therapy as potential cure	Absolute contraindication	0 (0)
	Relative contraindication	20 (9.5)
	Not a contraindication	160 (76.2)
	Unsure	30 (14.3)
Relapsed disease following 1st CAR-T, now receiving 2nd CAR-T therapy	Absolute contraindication	13 (6.2)
	Relative contraindication	102 (48.6)
	Not a contraindication	64 (30.5)
	Unsure	31 (14.7)
Relapsed disease following 1st HCT, now receiving 2nd CAR-T therapy	Absolute contraindication	11 (5.2)
	Relative contraindication	82 (39.1)
	Not a contraindication	88 (41.9)
	Unsure	29 (13.8)
Relapsed disease following 2 or more HCT, now receiving CAR-T therapy	Absolute contraindication	56 (26.7)
	Relative contraindication	82 (39.1)
	Not a contraindication	43 (20.4)
	Unsure	29 (13.8)
Active neurotoxicity due to CAR-T	Absolute contraindication	29 (13.8)
	Relative contraindication	91 (43.3)
	Not a contraindication	60 (28.6)
	Unsure	30 (14.3)
Presence of MOF	Absolute contraindication	72 (34.3)
	Relative contraindication	83 (39.5)
	Not a contraindication	33 (15.7)
	Unsure	22 (10.5)
Active CRS or other CAR-T associated inflammatory syndrome	Absolute contraindication	8 (3.8)
	Relative contraindication	57 (27.1)
	Not a contraindication	119 (56.7)
	Unsure	26 (12.4)
Receipt of investigational phase 1 CAR-T product	Absolute contraindication	6 (2.9)
	Relative contraindication	45 (21.4)
	Not a contraindication	113 (53.8)
	Unsure	46 (21.9)

CAR-T, chimeric antigen receptor T-cell; ECMO, extracorporeal membrane oxygenation; HCT, hematopoietic cell transplant, MOF, multiple organ failure; CRS, cytokine release syndrome.

We identified 8 scenarios which may affect perceived ECMO candidacy and asked respondents to rank each as an absolute contraindication, relative contraindication, not a contraindication, or if they were unsure if it was a contraindication for ECMO use in CAR-T patients. The use of primary CAR-T therapy as a potential cure was not considered a contraindication for ECMO by 76.2% of respondents and was only considered a relative contraindication by 9.5% of respondents. Receipt of a second CAR-T therapy following disease relapse was considered an absolute contraindication (6.2%), relative contraindication (48.6%), and not a contraindication (30.5%) for ECMO support. The presence of multiple organ failure, active CAR-T therapy related ICANS, cytokine release syndrome (CRS)/CAR-T associated inflammatory syndrome, or receipt of an investigational phase I product was identified as an absolute contraindication for ECMO in 34.3%, 13.8%, 3.8%, and 2.9% of survey participants, respectively. Additionally, the presence of active CAR-T therapy related ICANS, multiple organ failure, CRS/CAR-T associated inflammatory syndrome, or receipt of an investigational phase I product was identified as a relative contraindication for ECMO in 43.3%, 39.5%, 27.1%, and 21.4% of survey participants, respectively. There were significant differences between how PCCM providers and non-PCCM providers considered the use of CAR-T therapy in various situations on ECMO candidacy (**Supplemental Table 2**).

Compared to HCT patients, providers were less likely to consider the presence of multiple organ failure in patients receiving CAR-T therapy as an absolute contraindication for ECMO (34.3% vs 54.3%; p<0.001). Providers were also less likely to consider relapsed disease following first HCT now receiving CAR-T therapy as an absolute contraindication for ECMO compared to the need for two or greater HCT (5.2 vs 17.6; p<0.001). However, more providers considered relapsed disease following two or more HCTs now receiving CAR-T as an absolute contraindication for ECMO compared to the need for two or greater HCT (26.7 vs 17.6; p=0.026) (**Table 6**).

**Table 6:** Comparison of responses for extracorporeal membrane oxygenation contraindications between hematopoietic cell transplant and chimeric antigen receptor t-cell therapy.

Statement	Absolute Contraindication, N (%)	p-value	Relative Contraindication, N (%)	p-value
Autologous or Allogeneic HCT	1 (0.5)	1	17 (8.1)	0.606
First CAR-T for potential cure	0 (0)		20 (9.5)	
Receipt of ≥ 2 HCT	37 (17.6)	<0.001	55 (26.2)	0.005
Relapsed disease following first HCT, now receiving CAR-T therapy	11 (5.2)		82 (39.1)	
Receipt of ≥ 2 HCT	37 (17.6)	0.026	55 (26.2)	0.005
Relapsed disease following 2 or more HCT, now receiving CAR-T therapy	56 (26.7)		82 (39.1)	
Presence of MOF with HCT	114 (54.3)	<0.001	61 (29.1)	0.024
Presence of MOF with CAR-T	72 (34.3)		83 (39.5)	

HCT, hematopoietic cell transplant; CAR-T, chimeric antigen receptor T-cell; MOF, multiple organ failure.

## Discussion

Due to historically poor outcomes, HCT patients have generally been considered poor candidates and thereby excluded from consideration for ECMO support (1–3, 9). However, these reports have some significant limitations when used to dictate clinical practice. Specifically, these retrospective reports contain insufficient patient- and treatment- specific data in a vastly heterogenous patient population, rely on databases with limited reliability of reporting, and are a compilation of data across multiple treatment eras. Interval advancements in both peri-HCT support and critical care therapies have resulted in improved patient outcomes for pediatric HCT patients who require intensive care and critical care resources (13, 14, 32). This is supported by a recent report of the ELSO database by Olson, et al, which showed an improvement in survival to hospital discharge in pediatric HCT patient requiring ECMO from 3% between 1991 and 2009 to 26% between 2010 and 2019 (21).

Reflective of the improved patient survival within the last decade, we found that the vast majority (99.5%) of survey respondents would consider pediatric HCT patients as candidates for ECMO, with only 0.5% considering it as an absolute contraindication. However, while there was consensus amongst respondents that pediatric HCT patients should generally be considered candidates for ECMO support, there were notable differences between provider groups on the role of specific patient and treatment related factors in evaluating for ECMO candidacy. PCCM designated more factors as absolute and relative contraindications for ECMO and selected all factors more frequently than non-PCCM providers. Significantly more PCCM providers considered an expected 1-year survival < 50% from the underlying disease, pre-engraftment, and two or greater HCT as absolute contraindications for ECMO than non-PCCM providers. The differences in response are not as apparent when comparing responses between other groups, such as institution location, institution HCT volume, institution ECMO volume, and the presence or absence of an institutional HCT-specific ECMO candidacy evaluation protocol (**Supplemental Tables 3–6**). In discussing whether ECMO should be offered to a pediatric HCT patient, 83.3% of respondents report that both the on-call ICU physician and a HCT (on-call or patient’s primary) physician contribute to the decision making though consensus between the teams is often variable. This highlights the differences between provider roles and emphasizes the importance of interdisciplinary collaboration when deciding on ECMO candidacy for a pediatric HCT patient.

While individual opinion is favorable for considering pediatric HCT patients as ECMO candidates, institutional practice is lagging as 6% of respondents reported that HCT is still considered an absolute contraindication for ECMO within their institution. When asked to identify an acceptable rate to hospital survival for HCT patients supported with ECMO, the majority (95.7%) of respondents selected a survival range ≤50%, which is lower than that of the overall pediatric ECMO population (33) and 49.1% responded that they would accept a rate of hospital survival between 20-30% for these patients. Interestingly, over the last decade, the survival to hospital discharge for pediatric HCT patients supported with ECMO has already achieved this level with 26% survival (21). This suggests that survival for these patients is at a generally acceptable level. Thus, we anticipate that access to ECMO for critically ill pediatric HCT patients will improve, similar to that of access to invasive mechanical ventilation, which was also historically considered futile, and become more widespread over time (32, 34). Currently, only 23% of respondents reported that their institution has a protocol or policy to guide the evaluation of ECMO for HCT patients. As ECMO support becomes more accepted and used in the pediatric HCT population, providers must also address clinical practice at an institutional or systematic level. The development of clinical guidelines for the evaluation of ECMO candidacy for these patients would allow for standardization of selection criteria, an essential aspect of improving the care and outcomes of this vulnerable patient population. To do so, many (39.5%) respondents would utilize consensus statements by medical societies/experts in the field. We therefore advocate to prioritize the formation of an international working group consisting of expert providers across a spectrum of specialties to discuss and provide consensus statements for the selection and care of pediatric patients undergoing treatment with HCT or other cellular therapy who may require ECMO support.

Respondents were less likely to exclude certain patients treated with CAR-T therapy for ECMO support compared to patients treated with HCT. For example, we found that respondents were significantly less likely to consider the presence of multiple organ failure as an absolute contraindication for ECMO for patients treated with CAR-T than for patients treated with HCT. We propose several factors that may contribute to this difference. CAR-T therapy comes with risk of significant toxicity, include CRS, which is a known complication of CAR-T which may result in critical illness and hemodynamic collapse. However, in general, such complications are manageable and limited to the initial few weeks after CAR-T infusion during the time of peak CAR-T expansion and activity (28, 29). In addition, pharmacologic agents such as tocilizumab, a monoclonal antibody against IL-6, have shown great promise in treating CAR-T related CRS, thereby providing both a potential treatment and endpoint for the clinical deterioration (35). Whereas, HCT-associated respiratory failure often has an uncertain etiology, thus making the reversibility difficult to discern. Additionally, historical data on ECMO use in patients treated with HCT showed dismal outcomes whereas there is no little to no data regarding ECMO outcomes for patients treated with CAR-T therapy. Although the current lack of data may limit the ability of providers to form evidence-based decisions, it allows providers to approach ECMO candidacy for CAR-T patients without the same biases as are held for the HCT population.

Our study has several important limitations. The survey was only distributed to PCCM and HCT/cellular therapy physicians practicing within the United States, Canada, and Europe and thus may not reflect a global perspective regarding ECMO candidacy and use for pediatric HCT and CAR-T patients, including those in resource limited settings. A significant majority of survey respondents reported that they work in an academic center (94.8%) or belong to at least one large research collaboration: PALISI, PALISI-HCT subgroup, ELSO, PBMTC (94.8%). As such, our results are skewed towards the opinions and practices of large academic centers which may not reflect those of physicians practicing in non-academic or smaller groups. Furthermore, we did not evaluate the responses of participants within the same institution for consensus regarding institutional ECMO practices. Therefore, an individual’s response regarding institutional practices is reflective of that individual’s interpretation of the institution’s practices, which may be biased and limit the generalizability of our results. We also did not differentiate opinions regarding the use veno-arterial and veno-venous ECMO, which carry different safety profiles. This may have significant implications when evaluating the risks and benefits of ECMO in an already high-risk population. Further, as there is currently no literature on ECMO outcomes for pediatric patients treated with CAR-T, the opinions presented likely reflect anecdotal or extrapolated experiences and should therefore be interpreted cautiously.

## Conclusion

As clinical outcomes in pediatric critical care medicine, HCT, and CAR-T therapy are evolving, so are the opinions and practices of physicians caring for patients in these fields. Our international survey serves as the first overall assessment of provider opinion and practice in the evaluation of ECMO candidacy for pediatric patients treated with HCT or CAR-T therapy. Our results reveal that most providers no longer consider all HCT as a contraindication for ECMO and that published outcomes for hospital survival in the last decade are at a generally acceptable level. However, institution practice is lagging and there remains important differences in how providers between disciplines approach ECMO candidacy evaluation. Our study highlights the need for multidisciplinary perspectives in collaborative efforts to publish clinical practice guidelines, establish institutional protocols, and improve candidacy selection. These measures will allow us to safely advance the use of ECMO in this vulnerable patient population.

## Data Availability Statement

The raw data supporting the conclusions of this article will be made available by the authors, without undue reservation.

## Author Contributions

SG, KF, JM, and HS contributed to the conceptualization of the study and drafted the survey. SG, KF, MN, AT, AS, RP, LL, GA, FW, JM, HS, and Scientific Review Committee of The Pediatric Acute Lung Injury and Sepsis Investigators (PALISI) Network contributed revisions and approved the final version of the survey. SG, KF, JM, MN, and the Pediatric Acute Lung Injury and Sepsis Investigators (PALISI) Network distributed the survey. SG and KF reviewed the data and contributed to the drafting and critical revisions of the manuscript. FW provided statistical analysis and contributed to the drafting of the manuscript. MN, AT, AS, RP, LL, GA, JM, HS, and Scientific Review Committee of The Pediatric Acute Lung Injury and Sepsis Investigators (PALISI) Network provided critical intellectual revisions of the content. All authors provided approval for the final version of the manuscript and agree to be accountable for all aspects of the work related to accuracy and integrity.

## Conflict of Interest

The authors declare that the research was conducted in the absence of any commercial or financial relationships that could be construed as a potential conflict of interest.

The reviewer NJS declared a shared affiliation with one of the authors JM, to the handling editor at the time of review.

## Publisher’s Note

All claims expressed in this article are solely those of the authors and do not necessarily represent those of their affiliated organizations, or those of the publisher, the editors and the reviewers. Any product that may be evaluated in this article, or claim that may be made by its manufacturer, is not guaranteed or endorsed by the publisher.
