# Supplementary material for: Extracorporeal Membrane Oxygenation Candidacy in Pediatric Patients Treated With Hematopoietic Stem Cell Transplant and Chimeric Antigen Receptor T-Cell Therapy: An International Survey
Source: Front Oncol. 2021 Dec 22;11:798236. doi: 10.3389/fonc.2021.798236 (PMC8727600; doi:10.3389/fonc.2021.798236)
Supplement: Supplementary file 1 [file DataSheet_1.pdf]

General Information

1) Indicate your current medical position:	○ Pediatric critical care physician<br>○ Pediatric bone marrow transplant physician<br>○ Oncologist care for patients receiving chimeric antigen receptor T-cell (CAR-T) therapy<br>○ Other (please specify): ______________________
2) Indicate the number of years of experience you have in the above position:	○ 0-5<br>○ 6-10<br>○ 11-20<br>○ >20
3) Are you the institutional ECMO director?	○ Yes<br>○ No
4) Your institution is a member of which of the following organizations? (check all that apply)	○ PALISI<br>○ PALISI HCT subgroup<br>○ ELSO<br>○ PBMTC<br>○ None of the above
5) What is your current institution?	○ ___________________________<br>○ Prefer not to answer
6) Where is your institution located?	○ United States<br>○ Canada<br>○ Europe
7) Which of the following best describes your institution?	○ Academic Center<br>○ Private practice<br>○ Government hospital<br>○ Other (please specify):_____________________________

Institution/Division Information

8) Does your institution provide medical care for HCT patients?	○ Yes<br>○ No<br>○ Unsure
9) In the last 5 years, approximately how many HCT were performed at your institution (per year)?	○ 0-10<br>○ 11-30<br>○ > 30<br>○ Unsure
10) In the last 5 years, approximately how many ECMO cases did your institution provide (per year)?	○ 0-10<br>○ 11-30<br>○ > 30<br>○ Unsure
11) Who participates in medical rounds on ECMO patients at your institution? (check all that apply)	○ Critical care physician<br>○ Consulting physicians<br>○ Surgeon<br>○ Pediatric critical care fellows<br>○ Pediatric residents<br>○ Advanced practice providers (NP/PA)<br>○ Other trainees<br>○ Unsure
12) In the last 5 years, approximately how many HCT patients were admitted to your PICU (per year)?	○ 0-20<br>○ 21-50<br>○ > 50<br>○ Unsure
13) Have any post-HCT patients received ECMO support at your institution in the last 5 years (or were transferred to another institution for ECMO)? If so, approximately how many (in total)?	○ No<br>○ Yes, 1-5<br>○ Yes, >5<br>○ Unsure

Opinions and Practices

14) Which of the following statements best fits your opinion regarding use of ECMO in pediatric HCT patients?	a) HCT is an absolute contraindication for ECMO and it should never be offered.<br>b) Every HCT patient is unique thus candidacy for ECMO should be considered on an individual basis.<br>c) ECMO criteria for HCT patients is the same as for any other patient
15) What statement best fits your institution's practice regarding use of ECMO in HCT patients?	a) HCT is an absolute contraindication for ECMO and it should never be offered.<br>b) Every HCT patient is unique thus candidacy for ECMO should be considered on an individual basis.<br>c) ECMO criteria for HCT patients is the same as for any other patient
16) Does your institution have a protocol and/or policy to decide ECMO candidacy for HCT patients?	a) Yes<br>b) No<br>c) Unsure
17) What source contributes the most to your opinion regarding ECMO candidacy in HCT patients?	a) Historical data on outcomes<br>b) Personal opinion based on past experience<br>c) Institutional experience<br>d) Current standard practice in region/country<br>e) Institutional policy/protocol<br>f) Other ___________________
18) In your opinion, an acceptable rate of survival to hospital discharge for HCT patients requiring ECMO is:	a) 20%-30%. (HCT patients comprise a special population therefore lower survival is acceptable)<br>b) 30- 40%. (Similar to survival for ECMO during CPR (E-CPR))<br>c) 40%-50%. (Similar to survival for ECMO due to pediatric sepsis)<br>d) 50% -60%. (Similar to survival for ECMO due to pediatric acute respiratory distress syndrome)<br>e) >60%
19) Which of the following initiatives would be the most helpful tool in establishing ECMO candidacy and criteria for HCT patients?	a) Case reports/series with acceptable outcomes<br>b) Registry reports showing acceptable outcomes<br>c) Consensus statement of experts/Medical societies<br>d) A clinical trial is necessary to answer this question<br>e) We do not need any further studies on this subject

20) The following factors are known to affect the outcomes of HCT patients. What factors do you consider absolute contraindication(s) for ECMO post-HCT (check all that apply):

- ○ Allogeneic HCT
- ○ Autologous HCT
- ○ Pre-engraftment
- ○ Secondary graft-failure
- ○ Non-oncologic disease as reason for transplant
- ○ Expected 1-year survival < 50%
- ○ Multiple organ failure
- ○ Graft versus host disease (GVHD), grade III or higher
- ○ Hepatic veno-occlusive disease/ sinusoid obstructive disease
- ○ Active pulmonary hemorrhage
- ○ Refractory thrombocytopenia
- ○ HCT<100 days
- ○ Mechanical ventilation > 14 days
- ○ Unknown etiology of decompensation
- ○ CAR-T
- ○ Unsure
- ○ Other (please specify):

21) The following factors are known to affect the outcomes of HCT patients. What factors do you consider relative contraindication(s) for ECMO post-HCT (check all that apply):

- ○ Allogeneic HCT
- ○ Autologous HCT
- ○ Pre-engraftment
- ○ Secondary graft-failure
- ○ Non-oncologic disease as reason for transplant
- ○ Expected 1-year survival < 50%
- ○ Multiple organ failure
- ○ Graft versus host disease (GVHD), grade III or higher
- ○ Hepatic veno-occlusive disease/ sinusoid obstructive disease
- ○ Active pulmonary hemorrhage
- ○ Refractory thrombocytopenia
- ○ HCT<100 days
- ○ Mechanical ventilation > 14 days
- ○ Unknown etiology of decompensation
- ○ CAR-T
- ○ Unsure
- ○ Other (please specify):

22) At your institution, the following physicians participate in the decision-making process for ECMO candidacy of HCT patients? (Choose all that apply)

a) ICU physician on-call/service
b) BMT physician on-call/service
c) Patient's primary BMT physician
d) ECMO director/ECMO consult team
e) Cannulating Surgeon
f) Other (Please specify): _______________

23) In your institution, how often is there consensus between the following medical teams regarding ECMO candidacy for an HCT patient?

a) Individual critical care physicians
○ Always ○ Sometimes ○ Never ○ Unsure

b) Critical care physician and BMT physician
○ Always ○ Sometimes ○ Never ○ Unsure

c) Critical care physician and cannulation surgeon
○ Always ○ Sometimes ○ Never ○ Unsure

d) Critical care physician and ECMO direct/consult team
○ Always ○ Sometimes ○ Never ○ Unsure

CAR-T

24) Does your center care for patients who have received CAR-T cell therapy?	a) Yes<br>b) No<br>c) Unsure
25) What statement best fits your opinion regarding use of ECMO in pediatric CAR-T patients?	a) ECMO for CAR-T and HCT should be evaluated similarly<br>b) ECMO for CAR-T and HCT are very different and should have evaluated individually<br>c) Undergoing treatment with CAR-T is an absolute contraindication for ECMO
26) What statement best fits your institution's practice regarding use of ECMO in CAR-T patients?	a) ECMO for CAR-T and HCT should be evaluated similarly<br>b) ECMO for CAR-T and HCT are very different and should have evaluated individually<br>c) Undergoing treatment with CAR-T is an absolute contraindication for ECMO<br>d) Unsure
27) What contributes the most to your opinion on ECMO candidacy in CAR-T patients?	a) Personal opinion based on past experience<br>b) Institutional experience<br>c) Current standard practice in region/country<br>d) Institutional policy/protocol<br>e) Other ___________________
28) What is your opinion regarding ECMO candidacy for CAR-T patients in each of the following circumstances (Likert Scale)-	
a) 1st CAR-T as a potential cure<br>○ Absolute contraindication ○ Relative contraindication ○ Not a contraindication ○ Unsure	
b) Relapsed disease following 1st CAR-T, now receiving 2nd CAR-T treatment<br>○ Absolute contraindication ○ Relative contraindication ○ Not a contraindication ○ Unsure	
c) Relapsed disease following first HCT, now receiving CAR-T<br>○ Absolute contraindication ○ Relative contraindication ○ Not a contraindication ○ Unsure	
d) Relapsed disease following 2 or more HCT, now receiving CAR-T<br>○ Absolute contraindication ○ Relative contraindication ○ Not a contraindication ○ Unsure	
e) Presence of active neurotoxicity due to CAR-T<br>○ Absolute contraindication ○ Relative contraindication ○ Not a contraindication ○ Unsure	
f) Presence of multiple organ dysfunction<br>○ Absolute contraindication ○ Relative contraindication ○ Not a contraindication ○ Unsure	
g) Active cytokine release syndrome (CRS) or other CAR-T associated inflammatory syndrome (ex: HLH/MAS)<br>○ Absolute contraindication ○ Relative contraindication ○ Not a contraindication ○ Unsure	
h) Receipt of investigational phase 1 CAR-T product as opposed to commercial product<br>○ Absolute contraindication ○ Relative contraindication ○ Not a contraindication ○ Unsure	

Supplementary Material Figure 1: Distributed survey questions and responses.
