# Supplementary material for: Extracorporeal Membrane Oxygenation Candidacy in Pediatric Patients Treated With Hematopoietic Stem Cell Transplant and Chimeric Antigen Receptor T-Cell Therapy: An International Survey
Source: Front Oncol. 2021 Dec 22;11:798236. doi: 10.3389/fonc.2021.798236 (PMC8727600; doi:10.3389/fonc.2021.798236)
Supplement: Supplementary file 2 [file DataSheet_2.pdf]

# of factors selected	Absolute Contraindication		Relative Contraindication	
	Median (IQR)	p-value	Median (IQR)	p-value
Overall	3 (2.75)		3 (2)	
PCCM	3 (3)	<0.001*	3 (2)	0.002*
Non-PCCM	2 (2)		2 (3)	
North American	3 (3)	0.193	3 (3)	0.086
Europe	4 (3)		3 (3)	
High volume ECMO	3 (5)	0.880	3 (2)	0.515
Low volume ECMO	3 (2.5)		3 (2)	
High volume HCT	2 (3)	0.395	3 (2)	0.363
Low volume HCT	3 (3)		3 (2)	
Institutional HCT ECMO protocol	3 (2.5)	0.416	3 (3)	0.225
No institutional HCT ECMO protocol	3 (3)		3 (2)	

Supplemental Table 1: Number of Selected Contraindications for the use of Extracorporeal Membrane Oxygenation for Patients Treated with Hematopoietic Cell Transplant. PCCM, pediatric critical care medicine; ECMO, extracorporeal membrane oxygenation, HCT hematopoietic cell transplant; IQR, interquartile range.
