# Supplementary material for: Extracorporeal Membrane Oxygenation Candidacy in Pediatric Patients Treated With Hematopoietic Stem Cell Transplant and Chimeric Antigen Receptor T-Cell Therapy: An International Survey
Source: Front Oncol. 2021 Dec 22;11:798236. doi: 10.3389/fonc.2021.798236 (PMC8727600; doi:10.3389/fonc.2021.798236)
Supplement: Supplementary file 3 [file DataSheet_3.pdf]

Question	Response	PCCM (N=159)<br>N (%)	Non-PCCM (N=51)<br>N (%)	p-value
What is your opinion regarding use of ECMO in pediatric CAR-T patients?	ECMO for CAR-T and HCT patients should be evaluated similarly	54 (34)	23 (45.1)	0.037*
	ECMO for CAR-T and HCT are very different and should have evaluated individually	104 (65.4)	28 (54.9)	
	Undergoing treatment with CAR-T is an absolute contraindication for ECMO	1 (0.6)	0 (0)	
What contributes the most to your opinion on ECMO candidacy in CAR-T patients?	Personal opinion based on past experience	38 (23.9)	15 (29.4)	0.768
	Institutional experience	40 (25.2)	11 (21.6)	
	Institutional policy/protocol	5 (3.1)	3 (5.9)	
	Current standard practice in region/country	59 (37.1)	16 (31.3)	
	Other	17 (10.7)	6 (11.8)	
What is your opinion regarding ECMO candidacy for CAR-T patients in each of the following circumstances				
1st CAR-T as a potential cure	Absolute Contraindication	0 (0)	0 (0)	0.008*
	Relative Contraindication	19 (11.9)	1 (1.9)	
	Not a contraindication	113 (71.1)	47 (92.2)	
	Unsure	27 (17)	3 (5.9)	
Relapsed disease following 1st CAR-T, now receiving 2nd CAR-T treatment	Absolute Contraindication	13 (8.2)	0 (0)	<0.001*
	Relative Contraindication	83 (52.2)	19 (37.2)	
	Not a contraindication	35 (22)	29 (56.9)	
	Unsure	28 (17.6)	3 (5.9)	
Relapsed disease following first HCT, now receiving CAR-T	Absolute Contraindication	11 (6.9)	0 (0)	<0.001*
	Relative Contraindication	71 (44.6)	11 (21.6)	
	Not a contraindication	51 (32.1)	37 (72.5)	
	Unsure	26 (16.4)	3 (5.9)	
Relapsed disease following 2 or more HCT, now receiving CAR-T	Absolute Contraindication	50 (31.4)	6 (11.8)	0.001*
	Relative Contraindication	57 (35.8)	25 (49)	
	Not a contraindication	26 (16.4)	17 (33.3)	
	Unsure	26 (16.4)	3 (5.9)	
Presence of active neurotoxicity due to CAR-T	Absolute Contraindication	24 (15.1)	5 (9.8)	0.002*
	Relative Contraindication	73 (45.9)	18 (35.3)	
	Not a contraindication	35 (22)	25 (49)	
	Unsure	27 (17)	3 (5.9)	
	Absolute Contraindication	58 (36.5)	14 (27.5)	0.477

Presence of multiple organ dysfunction	Relative Contraindication	59 (37.1)	24 (47.1)	
	Not a contraindication	24 (15.1)	9 (17.6)	
	Unsure	18 (11.3)	4 (7.8)	
Active cytokine release syndrome (CRS) or other CAR-T associated inflammatory syndrome (ex: HLH/MAS)	Absolute Contraindication	6 (3.8)	2 (3.9)	0.111
	Relative Contraindication	47 (29.5)	10 (19.6)	
	Not a contraindication	83 (52.2)	36 (70.6)	
	Unsure	23 (14.5)	3 (5.9)	
Receipt of investigational phase 1 CAR-T product as opposed to commercial product	Absolute Contraindication	6 (3.8)	0 (0)	0.003*
	Relative Contraindication	34 (21.4)	11 (21.6)	
	Not a contraindication	76 (47.8)	37 (72.5)	
	Unsure	43 (27)	3 (5.9)	

Supplemental Table 2: Comparison of responses between PCCM and non-PCCM providers regarding the use of ECMO for pediatric patients treated with CAR-T therapy. PCCM, pediatric critical care medicine; ECMO, extracorporeal membrane oxygenation; CAR-T, chimeric antigen receptor T-cell; HCT, hematopoietic cell transplant; HLH, hemophagocytic lymphohistiocytosis; MAS, macrophage activation syndrome
