# Supplementary material for: Extracorporeal Membrane Oxygenation Candidacy in Pediatric Patients Treated With Hematopoietic Stem Cell Transplant and Chimeric Antigen Receptor T-Cell Therapy: An International Survey
Source: Front Oncol. 2021 Dec 22;11:798236. doi: 10.3389/fonc.2021.798236 (PMC8727600; doi:10.3389/fonc.2021.798236)
Supplement: Supplementary file 4 [file DataSheet_4.pdf]

Factor	Absolute Contraindication N (%)	p-value	Relative Contraindication N (%)	p-value
Allogeneic HCT				
North America (N=191)	0 (0)	0.091	13 (6.8)	0.240
Europe (N=19)	1 (5.3)		0 (0)	
Autologous HCT				
North America	0 (0)	---	3 (1.6)	0.318
Europe	0 (0)		1 (5.3)	
≥ 2 HCT				
North America	32 (16.8)	0.297	54 (28.3)	0.030*
Europe	5 (26.3)		1 (5.3)	
Pre-engraftment				
North America	34 (17.8)	0.363	36 (18.8)	0.369
Europe	5 (26.3)		2 (10.5)	
Secondary graft failure				
North America	65 (34)	0.481	47 (24.6)	0.389
Europe	8 (42.1)		3 (15.8)	
Expected 1-year survival < 50% from underlying disease				
North America	69 (36.1)	0.157	62 (32.5)	0.307
Europe	10 (52.6)		4 (21.1)	
HCT < +100 days				
North America	6 (3.1)	0.109	30 (15.7)	0.546
Europe	2 (10.5)		4 (21.1)	
Non-oncologic disease as reason for transplant				
North America	0 (0)	---	7 (3.7)	0.396
Europe	0 (0)		0 (0)	
GVHD, grade III or higher				
North America	37 (19.4)	0.209	55 (28.8)	0.474
Europe	6 (31.6)		4 (21.1)	
VOD/SOS				
North America	35 (18.3)	0.784	49 (25.7)	0.950
Europe	3 (15.8)		5 (26.3)	
Active pulmonary hemorrhage				
North America	71 (37.2)	0.629	64 (33.5)	0.524
Europe	6 (31.6)		5 (26.3)	
Refractory thrombocytopenia				
North America	67 (35.1)	0.089	63 (33)	0.734
Europe	3 (15.8)		7 (36.8)	
MOF				
North America	99 (51.8)	0.024*	58 (30.4)	0.182
Europe	15 (78.9)		3 (15.8)	
Mechanical ventilation > 14 days				
North America	47 (24.6)	0.505	69 (36.1)	0.075
Europe	6 (31.6)		3 (15.8)	
Unknown etiology of decompensation				
North America	28 (14.7)	0.460	54 (28.3)	0.761
Europe	4 (21.1)		6 (31.6)	

Supplemental Table 3: Comparison of factors selected by respondents in North Americaversus Europe as absolute and relative contraindications for ECMO in pediatric patients treated with HCT. ECMO, extracorporeal membrane oxygenation; HCT, hematopoietic cell transplant; GVHD, graft versus host disease; VOD, veno-occlusive disease; SOS, sinusoidal obstruction syndrome; MOF, multiple organ failure
