# Supplementary material for: Extracorporeal Membrane Oxygenation Candidacy in Pediatric Patients Treated With Hematopoietic Stem Cell Transplant and Chimeric Antigen Receptor T-Cell Therapy: An International Survey
Source: Front Oncol. 2021 Dec 22;11:798236. doi: 10.3389/fonc.2021.798236 (PMC8727600; doi:10.3389/fonc.2021.798236)
Supplement: Supplementary file 5 [file DataSheet_5.pdf]

Factor	Absolute Contraindication N (%)	p-value	Relative Contraindication N (%)	p-value
Allogeneic HCT				
High-volume HCT Center (N=103)	0 (0)	0.270	9 (8.7)	0.210
Low-volume HCT Center (N=38)	1 (2.6)		1 (2.6)	
Autologous HCT				
High-volume HCT Center	0 (0)	---	1 (1)	0.468
Low-volume HCT Center	0 (0)		1 (2.6)	
≥ 2 HCT				
High-volume HCT Center	15 (14.6)	0.105	29 (28.2)	0.596
Low-volume HCT Center	10 (26.3)		9 (23.7)	
Pre-engraftment				
High-volume HCT Center	17 (16.5)	0.919	18 (17.5)	0.313
Low-volume HCT Center	6 (15.8)		4 (10.5)	
Secondary graft failure				
High-volume HCT Center	35 (34)	0.572	27 (26.2)	0.529
Low-volume HCT Center	11 (28.9)		8 (21.1)	
Expected 1-year survival < 50% from underlying disease				
High-volume HCT Center	28 (27.2)	0.023*	31 (30.1)	0.661
Low-volume HCT Center	18 (47.4)		10 (26.3)	
HCT < +100 days				
High-volume HCT Center	4 (3.9)	0.574	13 (12.6)	0.933
Low-volume HCT Center	0 (0)		5 (13.2)	
Non-oncologic disease as reason for transplant				
High-volume HCT Center	0 (0)	---	4 (3.9)	0.574
Low-volume HCT Center	0 (0)		0 (0)	
GVHD, grade III or higher				
High-volume HCT Center	18 (17.5)	0.406	29 (28.2)	0.485
Low-volume HCT Center	9 (23.7)		13 (34.2)	
VOD/SOS				
High-volume HCT Center	11 (10.7)	0.222	24 (23.3)	0.491
Low-volume HCT Center	7 (18.4)		11 (28.9)	
Active pulmonary hemorrhage				
High-volume HCT Center	37 (35.9)	0.920	27 (26.2)	0.218
Low-volume HCT Center	14 (36.8)		14 (36.8)	
Refractory thrombocytopenia				
High-volume HCT Center	31 (30.1)	0.865	32 (31.1)	0.722
Low-volume HCT Center	12 (31.6)		13 (34.2)	
MOF				
High-volume HCT Center	60 (58.3)	0.550	30 (29.1)	0.778
Low-volume HCT Center	20 (52.6)		12 (31.6)	
Mechanical ventilation > 14 days				
High-volume HCT Center	24 (23.3)	0.191	29 (28.2)	0.691
Low-volume HCT Center	13 (34.2)		12 (31.6)	
Unknown etiology of decompensation				
High-volume HCT Center	17 (16.5)	0.083	29 (28.2)	0.198
Low-volume HCT Center	2 (5.3)		15 (39.5)	

Supplemental Table 4: Comparison of factors selected by respondents in high volume (≥30/year) versus low volume (<30/year) HCT centers as absolute and relative contraindications for ECMO in pediatric patients treated with HCT. HCT, hematopoietic cell transplant; ECMO, extracorporeal membrane oxygenation; GVHD, graft versus host disease; VOD, veno-occlusive disease; SOS, sinusoidal obstruction syndrome; MOF, multiple organ failure
