# Supplementary material for: Extracorporeal Membrane Oxygenation Candidacy in Pediatric Patients Treated With Hematopoietic Stem Cell Transplant and Chimeric Antigen Receptor T-Cell Therapy: An International Survey
Source: Front Oncol. 2021 Dec 22;11:798236. doi: 10.3389/fonc.2021.798236 (PMC8727600; doi:10.3389/fonc.2021.798236)
Supplement: Supplementary file 6 [file DataSheet_6.pdf]

Factor	Absolute Contraindication N (%)	p-value	Relative Contraindication N (%)	p-value
Allogeneic HCT				
High-volume ECMO Center (N=66)	1 (1.5)	0.379	5 (7.6)	0.782
Low-volume ECMO Center (N=108)	0 (0)		7 (6.5)	
Autologous HCT				
High-volume ECMO Center	0 (0)	---	2 (3)	0.635
Low-volume ECMO Center	0 (0)		2 (1.9)	
≥ 2 HCT				
High-volume ECMO Center	13 (19.7)	0.503	11 (16.7)	0.072
Low-volume ECMO Center	17 (15.7)		31 (28.7)	
Pre-engraftment				
High-volume ECMO Center	15 (22.7)	0.502	16 (24.2)	0.165
Low-volume ECMO Center	20 (18.5)		17 (15.7)	
Secondary graft failure				
High-volume ECMO Center	27 (40.9)	0.163	9 (13.6)	0.073
Low-volume ECMO Center	33 (30.6)		27 (25)	
Expected 1-year survival < 50% from underlying disease				
High-volume ECMO Center	27 (40.9)	0.922	25 (37.9)	0.320
Low-volume ECMO Center	45 (41.7)		33 (30.6)	
HCT < +100 days				
High-volume ECMO Center	3 (4.5)	1	10 (15.2)	0.473
Low-volume ECMO Center	3 (2.8)		21 (19.4)	
Non-oncologic disease as reason for transplant				
High-volume ECMO Center	0 (0)	---	2 (3)	1
Low-volume ECMO Center	0 (0)		4 (3.7)	
GVHD, grade III or higher				
High-volume ECMO Center	14 (21.2)	0.766	16 (24.2)	0.608
Low-volume ECMO Center	25 (23.1)		30 (27.8)	
VOD/SOS				
High-volume ECMO Center	13 (19.7)	0.914	15 (22.7)	0.320
Low-volume ECMO Center	22 (20.4)		32 (29.6)	
Active pulmonary hemorrhage				
High-volume ECMO Center	23 (34.8)	0.512	22 (33.3)	0.709
Low-volume ECMO Center	43 (39.8)		39 (36.1)	
Refractory thrombocytopenia				
High-volume ECMO Center	26 (39.4)	0.232	20 (30.3)	0.365
Low-volume ECMO Center	33 (30.6)		40 (37)	
MOF				
High-volume ECMO Center	38 (57.6)	0.794	18 (27.3)	0.845
Low-volume ECMO Center	60 (55.6)		28 (25.9)	
Mechanical ventilation > 14 days				
High-volume ECMO Center	18 (27.3)	0.952	20 (30.3)	0.432
Low-volume ECMO Center	29 (26.9)		39 (36.1)	
Unknown etiology of decompensation				
High-volume ECMO Center	12 (18.2)	0.675	19 (28.8)	0.805
Low-volume ECMO Center	17 (15.7)		33 (30.6)	

Supplemental Table 5: Comparison of factors selected by respondents in high volume (≥30/year) versus low volume (<30/year) ECMO centers as absolute and relative contraindications for ECMO in pediatric patients treated with HCT. ECMO, extracorporeal membrane oxygenation; HCT, hematopoietic cell transplant; GVHD, graft versus host disease; VOD, veno-occlusive disease; SOS, sinusoidal obstruction syndrome; MOF, multiple organ failure
