# Supplementary material for: Extracorporeal Membrane Oxygenation Candidacy in Pediatric Patients Treated With Hematopoietic Stem Cell Transplant and Chimeric Antigen Receptor T-Cell Therapy: An International Survey
Source: Front Oncol. 2021 Dec 22;11:798236. doi: 10.3389/fonc.2021.798236 (PMC8727600; doi:10.3389/fonc.2021.798236)
Supplement: Supplementary file 7 [file DataSheet_7.pdf]

Factor	Absolute Contraindication N (%)	p-value	Relative Contraindication N (%)	p-value
Allogeneic HCT				
Institutional HCT ECMO protocol (N=48)	0 (0)	1	5 (10.4)	0.167
No institutional HCT ECMO protocol (N=162)	1 (0.6)		8 (4.9)	
Autologous HCT				
Institutional HCT ECMO protocol	0 (0)	---	2 (4.2)	0.225
No institutional HCT ECMO protocol	0 (0)		2 (1.2)	
≥ 2 HCT				
Institutional HCT ECMO protocol	7 (14.6)	0.530	14 (29.2)	0.593
No institutional HCT ECMO protocol	30 (18.5)		41 (25.3)	
Pre-engraftment				
Institutional HCT ECMO protocol	7 (14.6)	0.419	8 (16.7)	0.770
No institutional HCT ECMO protocol	32 (19.8)		30 (18.5)	
Secondary graft failure				
Institutional HCT ECMO protocol	20 (41.7)	0.253	15 (31.3)	0.168
No institutional HCT ECMO protocol	53 (32.7)		35 (21.6)	
Expected 1-year survival < 50% from underlying disease				
Institutional HCT ECMO protocol	18 (37.5)	0.985	20 (41.7)	0.082
No institutional HCT ECMO protocol	61 (37.7)		46 (28.4)	
HCT < +100 days				
Institutional HCT ECMO protocol	3 (6.3)	0.315	11 (22.9)	0.150
No institutional HCT ECMO protocol	5 (3.1)		23 (14.2)	
Non-oncologic disease as reason for transplant				
Institutional HCT ECMO protocol	0 (0)	---	3 (6.3)	0.198
No institutional HCT ECMO protocol	0 (0)		4 (2.5)	
GVHD, grade III or higher				
Institutional HCT ECMO protocol	12 (25)	0.377	14 (29.2)	0.851
No institutional HCT ECMO protocol	31 (19.1)		45 (27.8)	
VOD/SOS				
Institutional HCT ECMO protocol	11 (22.9)	0.323	14 (29.2)	0.533
No institutional HCT ECMO protocol	27 (16.7)		40 (24.7)	
Active pulmonary hemorrhage				
Institutional HCT ECMO protocol	20 (41.7)	0.413	15 (31.3)	0.787
No institutional HCT ECMO protocol	57 (35.2)		54 (33.3)	
Refractory thrombocytopenia				
Institutional HCT ECMO protocol	14 (29.2)	0.486	17 (35.4)	0.727
No institutional HCT ECMO protocol	56 (34.6)		53 (32.7)	
MOF				
Institutional HCT ECMO protocol	27 (56.3)	0.756	19 (39.6)	0.067
No institutional HCT ECMO protocol	87 (53.7)		42 (25.9)	
Mechanical ventilation > 14 days				
Institutional HCT ECMO protocol	16 (33.3)	0.142	20 (41.7)	0.220
No institutional HCT ECMO protocol	37 (22.8)		52 (32.1)	
Unknown etiology of decompensation				
Institutional HCT ECMO protocol	10 (20.8)	0.219	13 (27.1)	0.795
No institutional HCT ECMO protocol	22 (13.6)		47 (29)	

Supplemental Table 6: Comparison of factors selected by respondents in centers with and without a specific HCT ECMO protocol as absolute and relative contraindications for ECMO in pediatric patients treated with HCT. HCT, hematopoietic cell transplant; ECMO, extracorporeal membrane oxygenation; GVHD, graft versus host disease; VOD, veno-occlusive disease; SOS, sinusoidal obstruction syndrome; MOF, multiple organ failure
